# Supplementary material for: Sequence and structural patterns detected in entangled proteins reveal the importance of co-translational folding
Source: Sci Rep. 2019 Jun 10;9:8426. doi: 10.1038/s41598-019-44928-3 (PMC6557820; doi:10.1038/s41598-019-44928-3)
Supplement: Supplementary file 1 — Supplementary Information [file 41598_2019_44928_MOESM1_ESM.pdf]

Sequence and structural patterns detected in entangled proteins reveal the importance of co-translational folding

Marco Baiesi[1,2], Enzo Orlandini[1,2], Flavio Seno[1,2,*], and Antonio Trovato[1,2]

[1]Department of Physics and Astronomy, University of Padova, Via Marzolo 8, I-35131 Padova, Italy
[2]INFN, Sezione di Padova, Via Marzolo 8, I-35131 Padova, Italy
[*]seno@pd.infn.it

ABSTRACT

This Supplementary Information contains additional figures that complement those in the main text.

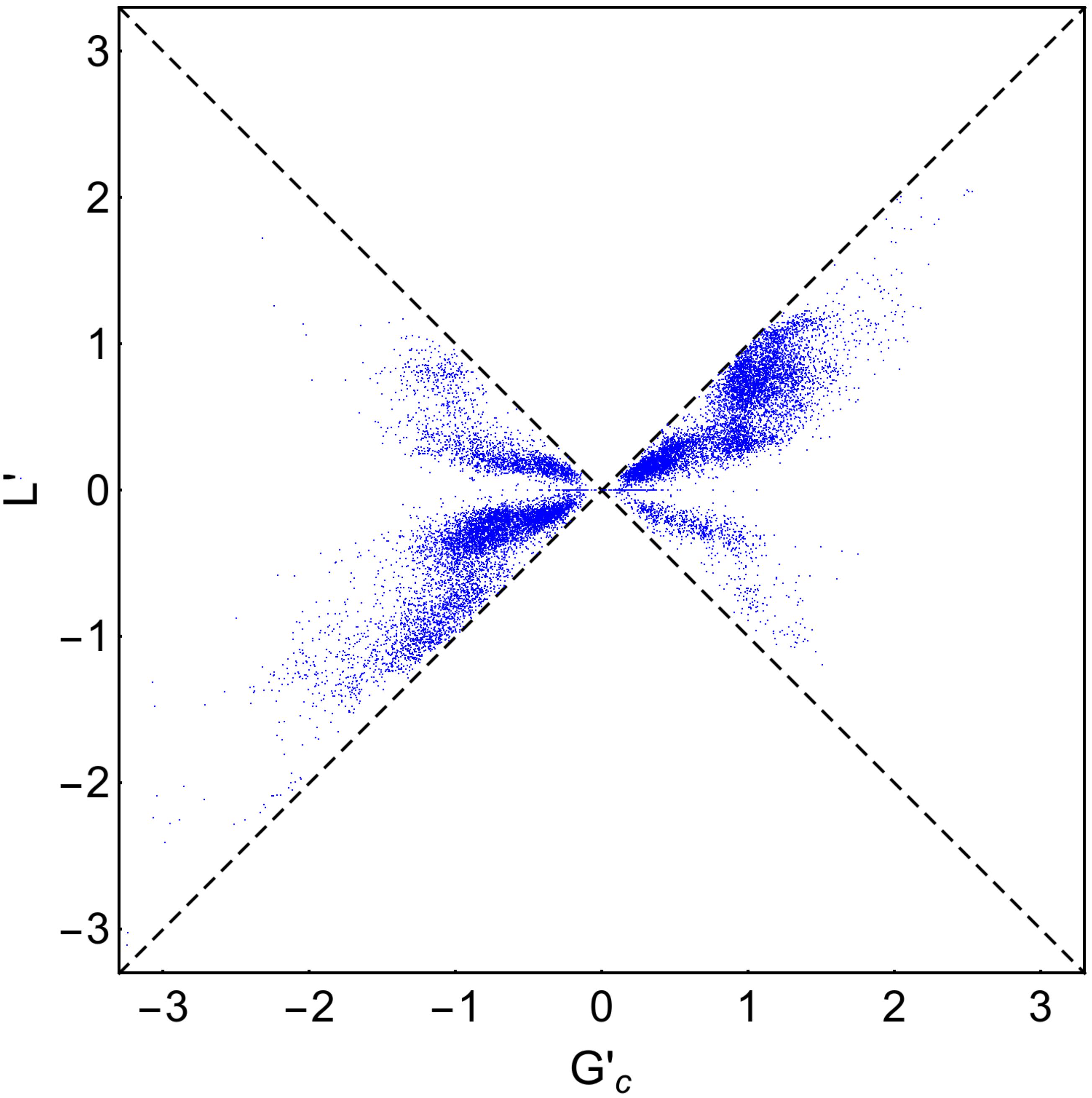

Figure S1. Enlarged view of the linking entanglement vs. the Gaussian entanglement. Each point represents a protein in the CATH database. Clusters of data are visible.

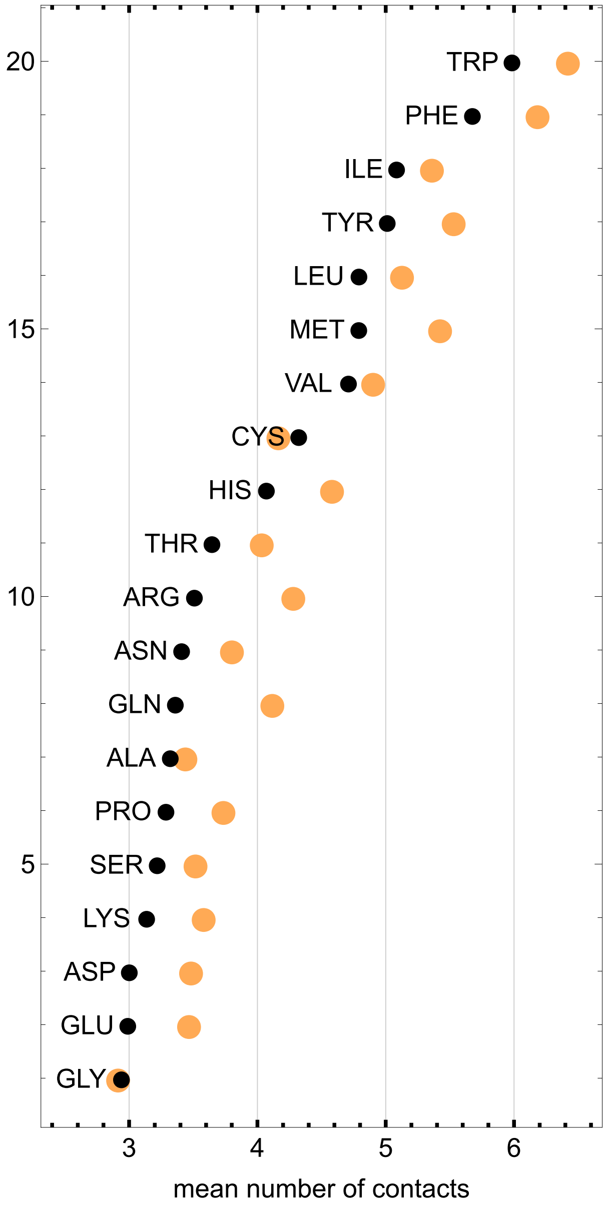

Figure S2. Amino acids ranked according to the average number of contacts (black dots) that they form with other amino acids in the protein structures of the CATH database. We recall that a contact between amino acids a and b is defined as a configuration where any of the heavy atoms of a is at distance lower than 4.5 from another heavy atom of b. The orange dots represent the average number of contacts of amino acids at the end of entangled loops. One can note that these are typically larger than the standard average values.

	TRP	PHE	TYR	CYS	ILE	VAL	LEU	MET	HIS	THR	ARG	PRO	GLN	ASN	ALA	SER	LYS	GLY	GLU	ASP
TRP	−31	−5	−29	−7	0	23	1	−20	−40	4	−29	−46	−40	−33	−16	3	−12	−25	−5	−23
PHE	−5	−9	−12	26	15	13	7	8	−10	3	−11	−39	−10	−11	11	11	−2	8	−18	−17
TYR	−29	−12	−21	36	4	4	4	−16	−32	−5	−21	−40	−20	−36	−16	−4	−20	−31	−34	−30
CYS	−7	26	36	130	49	56	52	15	−1	54	−9	−56	−11	6	42	25	49	52	6	54
ILE	0	15	4	49	50	49	37	21	11	32	8	−21	2	2	24	20	12	10	9	26
VAL	23	13	4	56	49	54	36	30	−1	34	14	−16	4	4	38	28	29	21	15	27
LEU	1	7	4	52	37	36	20	18	−10	16	−14	−21	0	−10	15	10	−12	−1	−2	−1
MET	−20	8	−16	15	21	30	18	21	−35	6	−22	−35	−23	−45	3	−1	−24	10	−35	−22
HIS	−40	−10	−32	−1	11	−1	−10	−35	9	−23	−3	−33	−29	−8	−10	−16	−2	−23	−21	−22
THR	4	3	−5	54	32	34	16	6	−23	17	−14	−32	−6	−9	15	6	10	12	−10	8
ARG	−29	−11	−21	−9	8	14	−14	−22	−3	−14	−42	−54	−23	−39	−22	−12	−21	−30	−29	−26
PRO	−46	−39	−40	−56	−21	−16	−21	−35	−33	−32	−54	−49	−46	−46	−20	−25	−32	−23	−42	−28
GLN	−40	−10	−20	−11	2	4	0	−23	−29	−6	−23	−46	−18	−35	−25	−23	−14	−30	−12	−10
ASN	−33	−11	−36	6	2	4	−10	−45	−8	−9	−39	−46	−35	−9	−16	−20	−28	−31	−41	−21
ALA	−16	11	−16	42	24	38	15	3	−10	15	−22	−20	−25	−16	−6	1	−3	−26	−12	3
SER	3	11	−4	25	20	28	10	−1	−16	6	−12	−25	−23	−20	1	12	2	−7	−15	−5
LYS	−12	−2	−20	49	12	29	−12	−24	−2	10	−21	−32	−14	−28	−3	2	−6	−20	−17	−22
GLY	−25	8	−31	52	10	21	−1	10	−23	12	−30	−23	−30	−31	−26	−7	−20	−29	−27	−6
GLU	−5	−18	−34	6	9	15	−2	−35	−21	−10	−29	−42	−12	−41	−12	−15	−17	−27	−37	−28
ASP	−23	−17	−30	54	26	27	−1	−22	−22	8	−26	−28	−10	−21	3	−5	−22	−6	−28	−38

Figure S3. Enrichment score ΔE_{enr} for entangled contacts, as in Fig. 7B of the main text but with a higher threshold $|G'_c(i)| > 1.2$ defining entangled loops.

	TRP	PHE	TYR	CYS	ILE	VAL	LEU	MET	HIS	THR	ARG	PRO	GLN	ASN	ALA	SER	LYS	GLY	GLU	ASP
TRP	−28	−12	−19	12	−5	20	−4	−13	−33	0	−17	−42	−29	−23	−10	−6	−22	−20	−13	−8
PHE	−12	−11	−12	25	14	18	−2	−7	−18	2	−14	−28	−12	−15	−1	10	−4	8	−16	−6
TYR	−19	−12	−5	29	5	8	−2	−11	−22	−2	−13	−32	−14	−22	−14	0	−10	−14	−26	−19
CYS	12	25	29	74	45	48	36	5	9	37	−17	−44	0	23	18	32	21	48	21	69
ILE	−5	14	5	45	35	37	18	7	12	20	3	−14	−4	1	9	19	8	7	9	34
VAL	20	18	8	48	37	50	22	11	6	28	17	−9	7	15	27	23	23	23	13	32
LEU	−4	−2	−2	36	18	22	4	−5	−8	1	−14	−26	−8	−5	0	−5	−12	0	−9	4
MET	−13	−7	−11	5	7	11	−5	−5	−18	−8	−25	−30	−26	−26	−10	−6	−20	−2	−28	0
HIS	−33	−18	−22	9	12	6	−8	−18	−5	−13	−8	−32	−22	0	−4	−9	3	−4	−12	−11
THR	0	2	−2	37	20	28	1	−8	−13	20	−4	−21	−1	−8	−3	6	13	13	2	15
ARG	−17	−14	−13	−17	3	17	−14	−25	−8	−4	−15	−37	−24	−20	−16	−10	−12	−22	−24	−14
PRO	−42	−28	−32	−44	−14	−9	−26	−30	−32	−21	−37	−43	−38	−32	−21	−28	−21	−10	−39	−15
GLN	−29	−12	−14	0	−4	7	−8	−26	−22	−1	−24	−38	−15	−17	−19	−11	−6	−14	−10	−13
ASN	−23	−15	−22	23	1	15	−5	−26	0	−8	−20	−32	−17	1	−9	−1	−16	−15	−23	−9
ALA	−10	−1	−14	18	9	27	0	−10	−4	−3	−16	−21	−19	−9	−13	−4	−3	−10	−15	15
SER	−6	10	0	32	19	23	−5	−6	−9	6	−10	−28	−11	−1	−4	11	−4	5	−8	−1
LYS	−22	−4	−10	21	8	23	−12	−20	3	13	−12	−21	−6	−16	−3	−4	4	−10	−17	−6
GLY	−20	8	−14	48	7	23	0	−2	−4	13	−22	−10	−14	−15	−10	5	−10	1	−15	14
GLU	−13	−16	−26	21	9	13	−9	−28	−12	2	−24	−39	−10	−23	−15	−8	−17	−15	−16	−13
ASP	−8	−6	−19	69	34	32	4	0	−11	15	−14	−15	−13	−9	15	−1	−6	14	−13	−2

Figure S4. Enrichment score ΔE_{enr} for entangled contacts, as in Fig. 7B of the main text but with a minimum separation of $s_0 = 5$ amino acid bonds between the thread and the loop.

	TRP	PHE	TYR	CYS	ILE	VAL	LEU	MET	HIS	THR	ARG	PRO	GLN	ASN	ALA	SER	LYS	GLY	GLU	ASP
TRP	–111	–111	–91	–98	–91	–79	–91	–88	–58	–33	–43	–46	–32	–14	–27	–22	–16	–2	–11	2
PHE	–111	–120	–89	–89	–106	–93	–102	–96	–39	–28	–22	–16	–9	6	–36	–4	5	7	21	32
TYR	–91	–89	–66	–66	–75	–64	–68	–67	–46	–19	–27	–30	–12	–1	–14	–2	–10	12	–3	2
CYS	–98	–89	–66	–194	–71	–66	–60	–60	–49	–21	–6	–7	6	4	–10	0	18	8	37	35
ILE	–91	–106	–75	–71	–113	–98	–102	–77	–18	–24	–1	9	6	27	–32	12	18	27	35	51
VAL	–79	–93	–64	–66	–98	–93	–86	–65	–13	–22	3	12	11	28	–25	14	16	29	35	51
LEU	–91	–102	–68	–60	–102	–86	–99	–71	–8	–10	4	13	16	36	–21	24	26	39	47	62
MET	–88	–96	–67	–60	–77	–65	–71	–72	–20	–10	6	5	11	21	–8	17	29	25	42	48
HIS	–58	–39	–46	–49	–18	–13	–8	–20	–53	–7	13	12	16	10	36	4	39	35	8	–3
THR	–33	–28	–19	–21	–24	–22	–10	–10	–7	1	16	32	20	23	33	27	29	47	42	44
ARG	–43	–22	–27	–6	–1	3	4	6	13	16	37	30	21	24	52	32	63	50	–19	–16
PRO	–46	–16	–30	–7	9	12	13	5	12	32	30	31	33	33	53	46	61	55	65	65
GLN	–32	–9	–12	6	6	11	16	11	16	20	21	33	29	29	61	40	39	59	59	59
ASN	–14	6	–1	4	27	28	36	21	10	23	24	33	29	12	63	35	42	46	54	41
ALA	–27	–36	–14	–10	–32	–25	–21	–8	36	33	52	53	61	63	37	61	69	73	90	95
SER	–22	–4	–2	0	12	14	24	17	4	27	32	46	40	35	61	45	51	61	60	54
LYS	–16	5	–10	18	18	16	26	29	39	29	63	61	39	42	69	51	76	66	2	3
GLY	–2	7	12	8	27	29	39	25	35	47	50	55	59	46	73	61	66	65	89	73
GLU	–11	21	–3	37	35	35	47	42	8	42	–19	65	59	54	90	60	2	89	113	114
ASP	2	32	2	35	51	51	62	48	–3	44	–16	65	59	41	95	54	3	73	114	95

	TRP	PHE	TYR	CYS	ILE	VAL	LEU	MET	HIS	THR	ARG	PRO	GLN	ASN	ALA	SER	LYS	GLY	GLU	ASP
TRP	–34	–15	–21	11	–3	21	0	–18	–49	0	–19	–42	–33	–22	–12	–6	–16	–24	–15	–22
PHE	–15	–9	–12	18	15	19	3	–8	–18	2	–14	–31	–16	–24	–1	6	–5	–1	–10	–10
TYR	–21	–12	–3	18	7	13	1	–9	–22	2	–14	–32	–16	–23	–11	0	–8	–20	–19	–18
CYS	11	18	18	72	38	44	32	14	12	24	–10	–55	–2	29	21	32	29	24	23	50
ILE	–3	15	7	38	38	40	22	9	9	17	9	–21	–2	–7	11	19	14	7	17	33
VAL	21	19	13	44	40	55	28	15	6	28	20	–9	6	10	29	26	29	18	23	28
LEU	0	3	1	32	22	28	10	–1	–7	4	–9	–25	–6	–5	4	1	–3	–2	–1	5
MET	–18	–8	–9	14	9	15	–1	–11	–15	–8	–26	–31	–28	–29	–8	–12	–20	–11	–23	–6
HIS	–49	–18	–22	12	9	6	–7	–15	–7	–16	–19	–36	–27	–11	–11	–15	–5	–12	–18	–17
THR	0	2	2	24	17	28	4	–8	–16	20	–6	–21	4	–10	–1	7	14	–6	8	13
ARG	–19	–14	–14	–10	9	20	–9	–26	–19	–6	–16	–39	–23	–24	–13	–10	–12	–31	–20	–20
PRO	–42	–31	–32	–55	–21	–9	–25	–31	–36	–21	–39	–40	–39	–35	–24	–28	–23	–25	–34	–20
GLN	–33	–16	–16	–2	–2	6	–6	–28	–27	4	–23	–39	–13	–20	–14	–4	–3	–26	–11	–16
ASN	–22	–24	–23	29	–7	10	–5	–29	–11	–10	–24	–35	–20	–1	–13	–6	–20	–28	–26	–17
ALA	–12	–1	–11	21	11	29	4	–8	–11	–1	–13	–24	–14	–13	–15	–5	–1	–23	–10	9
SER	–6	6	0	32	19	26	1	–12	–15	7	–10	–28	–4	–6	–5	15	0	–4	–8	–4
LYS	–16	–5	–8	29	14	29	–3	–20	–5	14	–12	–23	–3	–20	–1	0	2	–17	–15	–12
GLY	–24	–1	–20	24	7	18	–2	–11	–12	–6	–31	–25	–26	–28	–23	–4	–17	–10	–14	0
GLU	–15	–10	–19	23	17	23	–1	–23	–18	8	–20	–34	–11	–26	–10	–8	–15	–14	–16	–19
ASP	–22	–10	–18	50	33	28	5	–6	–17	13	–20	–20	–16	–17	9	–4	–12	0	–19	–16

Figure S5. Normal potential E_{norm} (top) and enrichment score ΔE_{enr} (bottom) for entangled contacts, with a minimum loop length $m_0 = 6$ instead of the value $m_0 = 10$ used in the main text (Fig. 7).
